# Dominant cixiid vector and transmission of ‘*Candidatus* Arsenophonus phytopathogenicus’ and ‘*Candidatus* Phytoplasma solani’-related strain 16SrXII-P in sugar beet in Austria

**DOI:** 10.1038/s41598-025-07035-0

**Published:** 2025-07-02

**Authors:** Christoph Kreitzer, Jelena Stepanović, Nikola Stanojević, Anna Rohringer, Marion Seiter, Emil Rekanović, Bojan Duduk

**Affiliations:** 1 AGRANA Research and Innovation Center GmbH, Tulln, 3430 Austria; 2 Institute of Pesticides and Environmental Protection, Laboratory of Phytopathology, Belgrade, 11080 Serbia

**Keywords:** Plant sciences, Entomology, Bacteria, Pathogens

## Abstract

The presence of fastidious phytopathogenic bacteria ‘*Candidatus* Arsenophonus phytopathogenicus’ and ‘*Candidatus* Phytoplasma solani’ challenges sugar beet cultivation in Austria. *Reptalus artemisiae* (Becker, 1865) (referred before as *Reptalus quinquecostatus sensu* Holzinger et al. 2003) is the principal vector of ‘*Ca*. P. solani’ to sugar beet in Serbia, while *Pentastiridius leporinus* is the primary vector of ‘*Ca*. A. phytopathogenicus’ in western Europe and can also transmit ‘*Ca*. P. solani’. This study investigates the epidemiology of the associated diseases in Austrian sugar beet fields. An extensive survey in 2024 at 33 locations revealed significant spatial and temporal variability in cixiid populations, with *R. artemisiae* emerging as the predominant species. Transmission trials and field surveys confirmed *R. artemisiae* as a vector of ‘*Ca*. A. phytopathogenicus’ and ‘*Ca*. P. solani’ 16SrXII-P driving a disease outbreak in Nikitsch. The predominance of *R. artemisiae* in nearly all monitored fields suggests its significant role in the pathogens’ epidemiology on a broader scale in the Pannonian plain. Spatial and temporal variations in pathogen incidence in Austrian sugar beet mirror patterns in Serbia, driven by *R. artemisiae*. The co-occurrence of ‘*Ca*. A. phytopathogenicus’ and ‘*Ca*. P. solani’ 16SrXII-A and 16SrXII-P and their transmission by two vectors complicate disease management efforts.

**Supplementary Information:**

The online version contains supplementary material available at 10.1038/s41598-025-07035-0.

## Introduction

Two plant pathogenic bacteria ‘*Candidatus* Arsenophonus phytopathogenicus’ and ‘*Candidatus* Phytoplasma solani’ 16SrXII-A, both vector-borne, phloem-limited obligate pathogens, have been associated with two devastating diseases of sugar beet in Europe, namely syndrome “Basses Richesses” (SBR) and rubbery taproot disease (RTD), respectively^1–4^. As the sugar beet cultivation area in Europe covers more than 2.8 Mha (FAOSTAT), economic loss due to these diseases can be significant. Substantial yield losses which threaten the profitability of the sugar beet industry in some parts of Germany as well as economic losses of more than 50% in France have both been attributed to SBR, while complete loss and unharvested sugar beet fields in Serbia and Slovakia have been attributed to RTD^3,5,6^. Though sporadic presence of ‘*Ca*. P. solani’ 16SrXII-A has been reported in SBR-affected sugar beets in France, ‘*Ca*. A. phytopathogenicus’ was initially named the SBR bacterium, as its major role in the disease had been documented^4^. Moreover, a ‘*Ca.* P. solani’-related strain 16SrXII-P has recently been described and shown to be the dominant strain in sugar beet in Germany, often present in mixed infection with ‘*Ca*. A. phytopathogenicus’^7,8^. The two diseases exhibit some degree of specificity in their symptoms, such as vascular discoloration in cross-sections of taproots and malformation of young leaves, which are associated with SBR, as opposed to rubbery taproots, wilting, and plant decline, which are associated with RTD. However, some symptoms are nonspecific, including the yellowing and proliferation of young leaves. Distinguishing between these symptoms in the field is challenging, as their expression depends on factors such as the time since infection, variable climate, and sugar beet variety, particularly in cases involving SBR-tolerant varieties and mixed infections^1,2,7,9,10^.

‘*Ca.* A. phytopathogenicus’ is a γ-proteobacterium and the single phytopathogenic member of genus *Arsenophonus* as others are endosymbionts of insects and other arthropods^11,12^. The presence of ‘*Ca.* A. phytopathogenicus’-associated SBR in sugar beet was reported in France, Germany and Switzerland, while in Germany ‘*Ca.* A. phytopathogenicus’ was also found in potato and onion^13–16^. Sporadic presence of ‘*Ca.* A. phytopathogenicus’ in sugar beet, usually in mixed infection with ‘*Ca.* P. solani’ 16SrXII-A has been reported in the Pannonian plain, where Austria suffers the highest incidence of the former pathogen^6^.

Phytoplasmas are pleomorphic, wall-less bacteria that are associated with hundreds of plant diseases. They are classified in a provisional taxon ‘*Candidatus* Phytoplasma’ within the class Mollicutes, where delineation of ‘*Ca*. Phytoplasma’ species is based on 16S rRNA nucleotide sequence similarity. A parallel classification system based on restriction fragment length polymorphism (RFLP) of the 16S rRNA gene also exists, with the designation of ribosomal groups and subgroups (16Sr group-subgroup)^17–19^. In the Pannonian plain as well as in France in Western Europe and in Russia in Eastern Europe, ‘*Ca.* P. solani’ 16SrXII-A has been reported in sugar beet, while in Germany, the recently described ‘*Ca.* P. solani’ 16SrXII-P has been revealed as the dominant strain^6–8^.

While experimental transmission of ‘*Ca.* A. phytopathogenicus’ to sugar beet by cixiid *Cixius wagneri* China has been reported in France, the only vector in central Europe on sugar beet and potato is the cixiid planthopper *Pentastiridius leporinus* Linnaeus^9,15^. However, *P. leporinus* is also considered to transmit ‘*Ca.* P. solani’-16SrXII-A and -P on sugar beet and potato^2,9,15,20^. On the other hand *Reptalus artemisiae* (Becker, 1865) is reported (under name *Reptalus quinquecostatus* Dufour *sensu* Holzinger et al.^21^) as the principal vector of ‘*Ca.* P. solani’ 16SrXII-A on sugar beet in the Pannonian plain^1,2,9,22–26^. Another cixiid, *Hyalesthes obsoletus* Signoret, which has been described as a prominent vector of ‘*Ca.* P. solani’ 16SrXII-A on several crops in Austria, is capable of infecting sugar beet as well^25,27^. Though the presence of ‘*Ca.* A. phytopathogenicus’ in *R. artemisiae* from Austria and Serbia has been documented (unpublished data), no data about its transmission is available.

In all central European countries, ‘*Ca.* P. solani’ dominates over ‘*Ca.* A. phytopathogenicus’ and in most of countries in the Pannonian plain the γ-proteobacterium has been detected only sporadically. Although Austria generally shares the same pathogen ratio with the rest of the Pannonian plain, ‘*Ca.* A. phytopathogenicus’ has been present more often than phytoplasma and in some localities even dominates the latter^6^. *Pentastiridius leporinus*, the principal vector of ‘*Ca.* A. phytopathogenicus’, has been reported in Austria, as well as *H. obsoletus* and *R. artemisiae*, vectors of ‘*Ca.* P. solani’ on sugar beet^27–29^.

This epidemiological study aimed to elucidate the roles of cixiids in the transmission of ‘*Ca*. A. phytopathogenicus’ and ‘*Ca*. P. solani’ to sugar beet in Austria. Consequently, experiments were conducted in 2024 on three sugar beet fields located in areas where both diseases were reported in the previous 2023 growing season^6^. To identify present and dominant vectors in sugar beet fields, surveys focused on planthopper cixiids were carried out across the sugar beet-cultivating area of Austria. Furthermore, transmission experiments were conducted to assess the ability of naturally infected *R. artemisiae*, the cixiid predominantly present in the fields, to transmit pathogens to sugar beet test plants under controlled conditions. Finally, the presence of disease in the experimental fields later in the season was correlated with the previously determined presence and infection of vectors.

## Results

### *Reptalus artemisiae* dominates cixiid communities across Austrian sugar beet fields

**Fig. 1:**
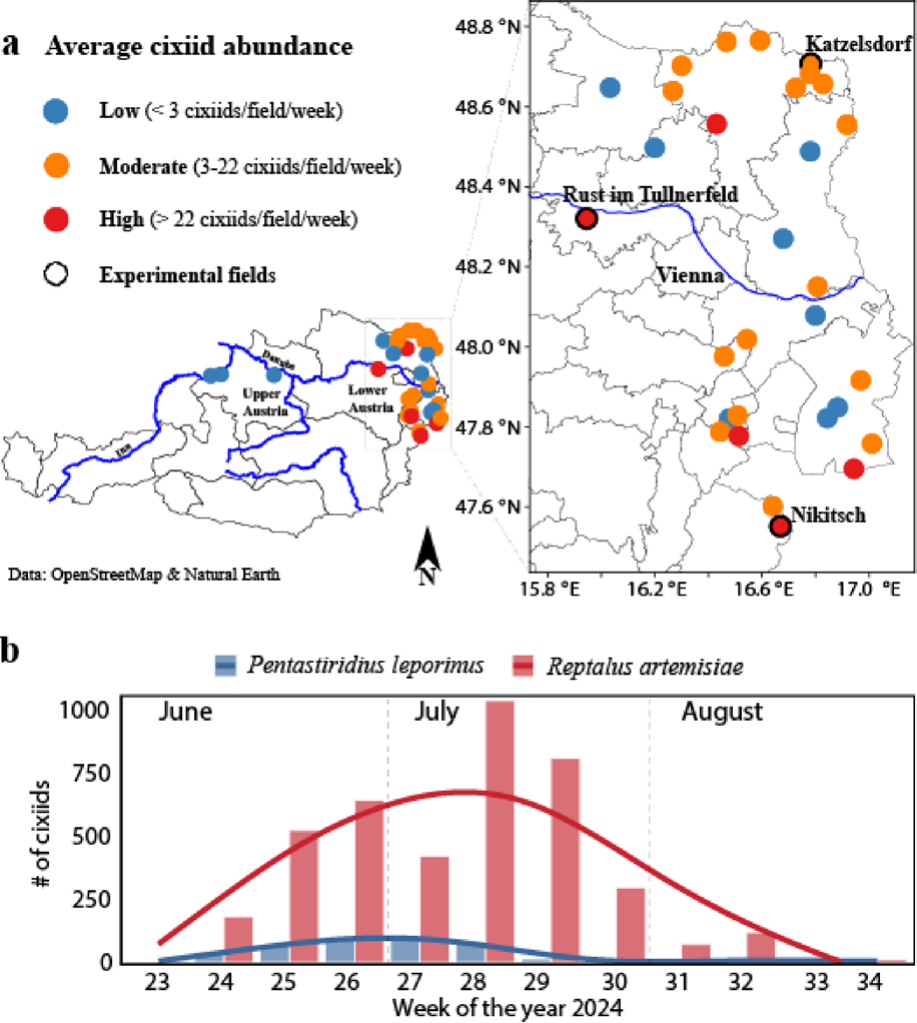
Cixiid monitoring sites across Austrian sugar beet fields and corresponding flight dynamics of adult* R. artemisiae* and* P. leporinus* specimens (**a**) Map of Austria depicting assessed fields and the average abundance of adult cixiids per sugar beet field according to the specified color pattern provided in the legend on the left. Specimens were caught using yellow sticky traps. The map was generated in R (version 4.3.2; R Core Team, 2023) with the packages ‘rnaturalearth’ (version 1.0.1) and ‘osmdata’ (version 0.2.5) (https://cran.r-project.org/) (**b**) Bar charts represent the total abundance (y-axis) of *R. artemisiae* (red) and *P. leporinus* (blue) adults stratified by week (x-axis) across all monitoring sites. Trend lines were fitted using a generalized additive model (GAM).

An extensive survey of cixiids was conducted from mid-June to the end of August 2024 at 33 locations across the sugar beet-cultivating regions of Austria (Fig. 1a, Supplementary Table S1. In total, 4,539 cixiids were captured using yellow sticky traps and examined morphologically. Based on external morphology, 403 specimens were identified as *P. leporinus*, 71 as *H. obsoletus*, and 4,065 as *Reptalus* sp. Of the 4,065 Reptalus specimens, 2,981 (73.3%) were male and subjected to genitalia examination. Among these, 2,968 (99.6%) were identified as *R. artemisiae*, and 13 (0.4%) as *R. panzeri*. Based on this male ratio, the remaining female *Reptalus* specimens were considered to occur in the same ratio as the males. The survey confirmed the presence of four cixiid species—all recognized vectors of fastidious plant pathogens—in commercial sugar beet fields, though their distribution varied across sites. *R. artemisiae*, *P. leporinus*, and *H. obsoletus* were detected at all surveyed sites in eastern Austria, the Pannonian Plain, the country’s primary sugar beet production area, whereas *R. panzeri* was recorded at only six locations.

Overall, *R. artemisiae* was the most abundant species, comprising 89.2% of all cixiids collected (4,048 out of 4,539 specimens, including identified males and proportionally assigned females), followed by *P. leporinus* at 8.9%, *H. obsoletus* at 1.6%, and *R. panzeri* at 0.3% (Supplementary Table S1). Cixiid abundance exhibited both spatial and temporal variation. For example, an average of 254 cixiids per sticky trap per week were captured in Nikitsch, compared to just four specimens per trap per week in Deutschkreutz, located 6 km away (Fig. 1a, Supplementary Table S1). Moreover, in the two locations that represent the far western sugar beet growing area in upper Austria (Moosham and Sankt Peter am Hart) and also depict the border to Bavaria Germany (Fig. 1a) only two specimens of *P. leporinus* were detected in total. Individuals of the three species were first detected in the beginning of the monitoring period, the first week of June (calendar week 23). The population of the most numerous species, *R. artemisiae* peaked in the second week of July (calendar week 28), while *P. leporinus* peaked about two weeks earlier, in the last week of June (calendar week 26) (Fig. 1b).

Three experimental fields (Fig. 1a) were selected for a comprehensive cixiid survey, molecular identification of *Reptalus* sp., testing of pathogen presence in insect, and transmission trials. These fields were chosen based on the presence of pathogens and associated disease symptoms on sugar beet in the previous 2023 growing season^6^. The fields are located in the far south (Nikitsch, Burgenland), central (Rust im Tullnerfeld, Lower Austria), and northern (Katzelsdorf, Lower Austria) parts of Austria (Fig. 1a; Table 1). In these fields, the cixiid population reflected the overall figures with three identified cixiid species, *R. panzeri* was not found. Among the three identified cixiid species *R. artemisiae* was the most abundant, representing 97.1% of all caught cixiids across the three locations. *Pentastiridius leporinus* was the second most abundant, accounting for 2.36%, whereas *H. obsoletus* was particularly scarce, with only 13 individuals found among 2361 total caught cixiids specimens during the entire monitoring period (Table 1). Morphological identification was confirmed, using molecular analyses based on different amplicon size of internal transcribed spacer 2 of the ribosomal DNA (ITS2), on a total of 140 specimens of *R. artemisiae* (110 from Nikitsch and 30 from Rust) as well as 35 specimens of *P. leporinus* (25 from Nikitsch and 10 from Rust) and all 15 *R. panzeri* from all six locations.

Insects used in transmission trials and for testing of pathogen presence were caught with sweeping nets. The numbers of insects caught by sweeping nets largely mirrored those from the sticky traps (Supplementary Table S1). During sweep netting on June 12 in Nikitsch and Rust only *R. artemisiae* was caught. The highest number of *R. artemisiae* specimens was detected in Nikitsch (~ 50 individuals per 10 sweeps), followed by Rust (~ 15 individuals per 10 sweeps). In Katzelsdorf the abundance of cixiids was particularly low; only four cixiids were collected in total (one *R. artemisiae* and three *P. leporinus*). Therefore, the transmission trial was not performed with cixiids from Katzelsdorf, but only with *R. artemisiae* from Nikitsch and Rust which were identified morphologically and subjected to molecular confirmation of the identification and pathogen detection.

### Three fastidious pathogens identified in *R. artemisiae*

Specimens of *R. artemisiae*, 20 specimens from each, Nikitsch and Rust, along with four cixiid specimens from Katzelsdorf (one *R. artemisiae* and three *P. leporinus*), were collected on July 12, using sweeping nets and subjected to morphological identification and molecular analyses. Additionally, the 110 and 30 specimens of *R. artemisiae* from yellow sticky traps in Nikitsch and Rust, respectively, and 25 and 10 specimens of *P. leporinus* from the same locations were included in the pathogen presence analysis, resulting in a total of 44 samples from sweeping nets and 175 from sticky traps. All *Reptalus* sp. male specimens from Nikitsch, Rust and the one from Katzelsdorf were morphologically identified as *R. artemisiae*, while molecular analyses of all 219 insect specimens collected on the three locations confirmed insect species identification, based on internal transcribed spacer 2 of the ribosomal DNA (ITS2).

Molecular analysis for pathogens presence revealed that none of the 50 *R. artemisiae* specimens nor any of the 10 *P. leporinus* collected in Rust, and four cixiids from Katzelsdorf, harbored either *‘Ca.* A. phytopathogenicus’ or *‘Ca.* P. solani’. However, in Nikitsch, out of 130 *R. artemisiae* specimens, 6.2% harbored *‘Ca.* A. phytopathogenicus’, while 4.7% harbored *‘Ca.* P. solani’ (3.1% harbored 16SrXII-P and 1.6% 16SrXII-A).

**Fig. 2:**
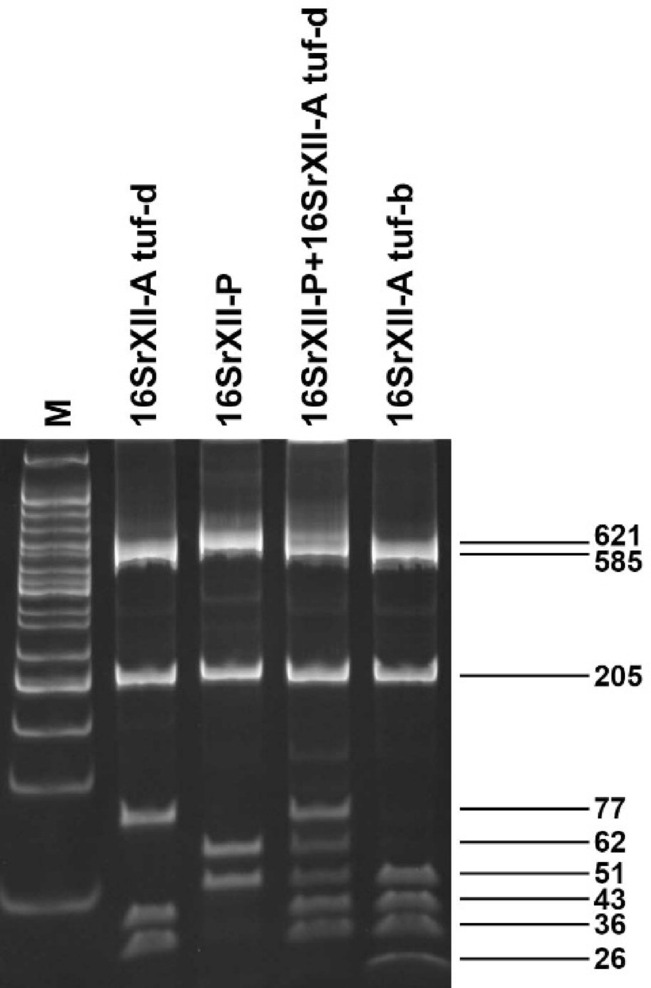
RFLP analysis using* HpyCH4IV* of the fTufAy/rTufAy amplicon showing representative profiles of ‘Ca. P. solani’ tuf types b, d (16SrXII-A), and 16SrXII-P from Austrian sugar beet fields. Cropped polyacrylamide gel (8%) showing representative restriction patterns of fTufAy/rTufAy amplicons of ‘Ca. P. solani’ strains 16SrXII-A tuf types b and d, as well as 16SrXII-P, obtained in this study, digested with the *HpyCH4*IV restriction enzyme. M, molecular marker CLS-MDNA-50BP Cleaver Scientific, fragment sizes of the obtained fragments are indicated in base pairs.

Out of 25 specimens of *P. leporinus* recovered from yellow sticky traps from Nikitsch, 28% harbored *‘Ca.* A. phytopathogenicus’, while 20% harbored *‘Ca.* P. solani’ (12% harbored 16SrXII-P and 8% 16SrXII-A). Although low in abundancy, *P. leporinus* has a higher pathogen infection rate than *R. artemisiae* − 28% *‘Ca.* A. phytopathogenicus’ and 20% *‘Ca.* P. solani’ compared to 6.2% and 4.7%, respectively (Table 1, Fig. 2). All *‘Ca.* P. solani’ 16SrXII-A strains detected in both *R. artemisiae* and *P. leporinus* from Nikitsch were characterized as tuf-d (Fig. 2).

**Table 1:** Cixiid planthoppers caught in sugar beet fields during the 2024 flight season and fastidious pathogens detected. As a locality where sugar beet plants expressed symptoms of fastidious pathogen infection later in the season, Nikitsch is highlighted in bold. * Three ‘*Ca*. A. phytopathogenicus’-positive *P. leporinus* specimens were in mixed infection, two with ‘*Ca*. P. solani’ 16SrXII-P and one with ‘*Ca*. P. solani’ 16SrXII-A.

Locality	GPS	Insects			Pathogens		
		Species	# caught	# tested	‘*Ca*. A. phytopathogenicus’	‘*Ca*. P. solani’	
						16SrXII-P	16SrXII-A
Rust im Tullnerfeld	48^o^19’14’’N 15^o^56’45’’E	*R. artemisiae*	529(94.4%)	50	0	0	0
		*P. leporinus*	26(4.8%)	10	0	0	0
		*H. obsoletus*	4(0.7%)	0	N/A	N/A	N/A
Katzelsdorf	48^o^42’24’’N 16^o^46’57’’E	*R. artemisiae*	10(22.7%)	1	0	0	0
		*P. leporinus*	31(70.5%)	3	0	0	0
		*H. obsoletus*	3(6.8%)	0	N/A	N/A	N/A
Nikitsch	47^o^33’06’’N 16^o^40’07’’E	*R. artemisiae*	1769(98.4%)	130	8(6.2%)	4(3.1%)	2(1.6%)
		*P. leporinus*	27(1.5%)	25	7(28%)*	3(12%)*	2(8%)*
		*H. obsoletus*	2(0.1%)	0	N/A	N/A	N/A

**Fig. 3:**
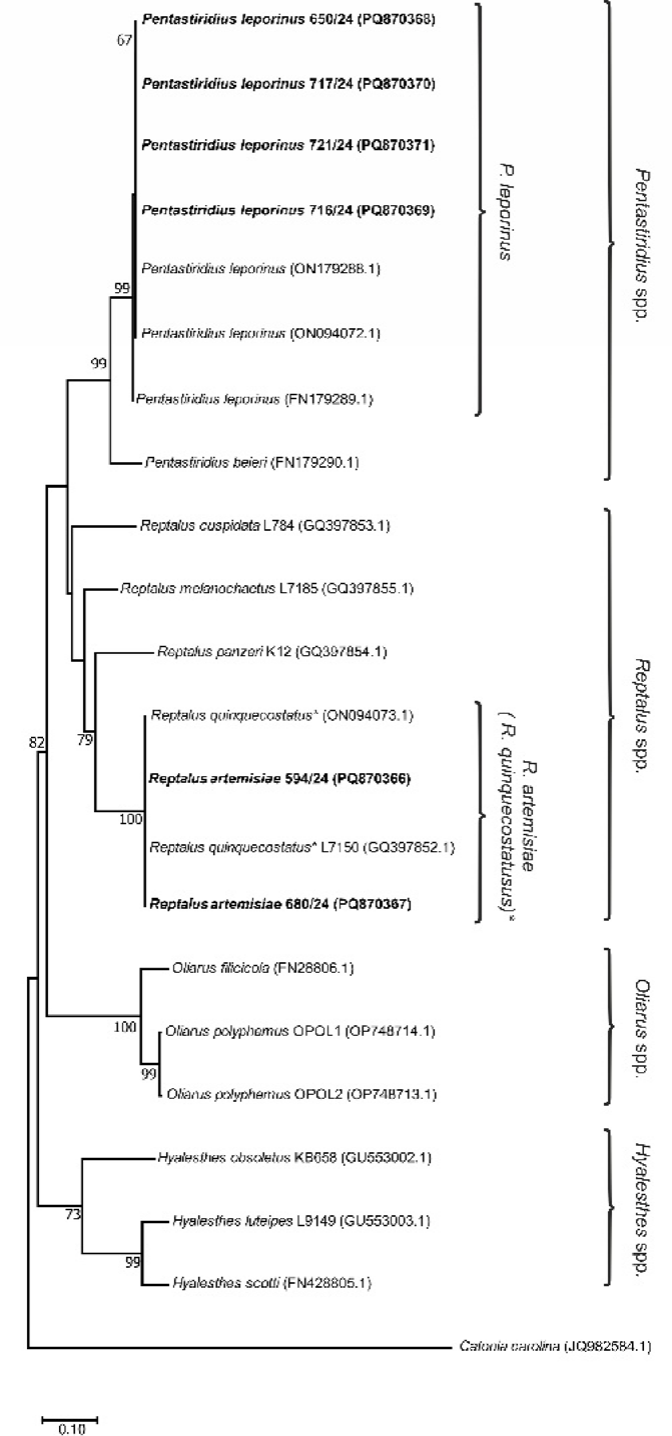
Maximum Likelihood phylogenetic analysis inferred from* COI* sequences obtained from cixiids in Austria and publicly available cixiid sequences. Maximum Likelihood phylogenetic tree inferred from 15 publicly available *COI* sequences of cixiids and six *COI* genotypes obtained in this study (four of *P. leporinus* and two of *R. artemisiae*). *Catonia carolina* (Achilidae) was used as an outgroup. GenBank accession numbers are noted in parentheses. Bootstrap support values (≥ 60) from 1,000 replicates, are provided adjacent to branches, with the scale showing substitutions per site. Sequences obtained in this research are highlighted in bold. * Downloaded *COI* sequences of cixiids referred in GenBank as *R. quinquecostatus* according to the old nomenclature.

To confirm identification of insect species harboring each pathogen, 14 *R. artemisiae* specimens, collected by sweep netting for the transmission trial, and four *P. leporinus* specimens, were subjected to *COI* sequencing. Analyses of the obtained *COI* sequences confirmed insect identification and revealed two genotypes among the 14 *R. artemisiae* specimens, which differed in a single nucleotide polymorphism (SNP). Each of the *P. leporinus* specimens represents a different genotype resulting in four genotypes that differ in no more than two SNPs. An ML phylogeny, constructed from *COI* sequences obtained from cixiids from sugar beet in Austria in the current study and publicly available sequences, resulted in several well-supported branches corresponding to the species. Notably, separate well-supported clusters of *R. artemisiae* (100) and *P. leporinus* (99) were obtained (Fig. 3).

### The presence of disease symptoms and pathogens in experimental fields

**Fig. 4:**
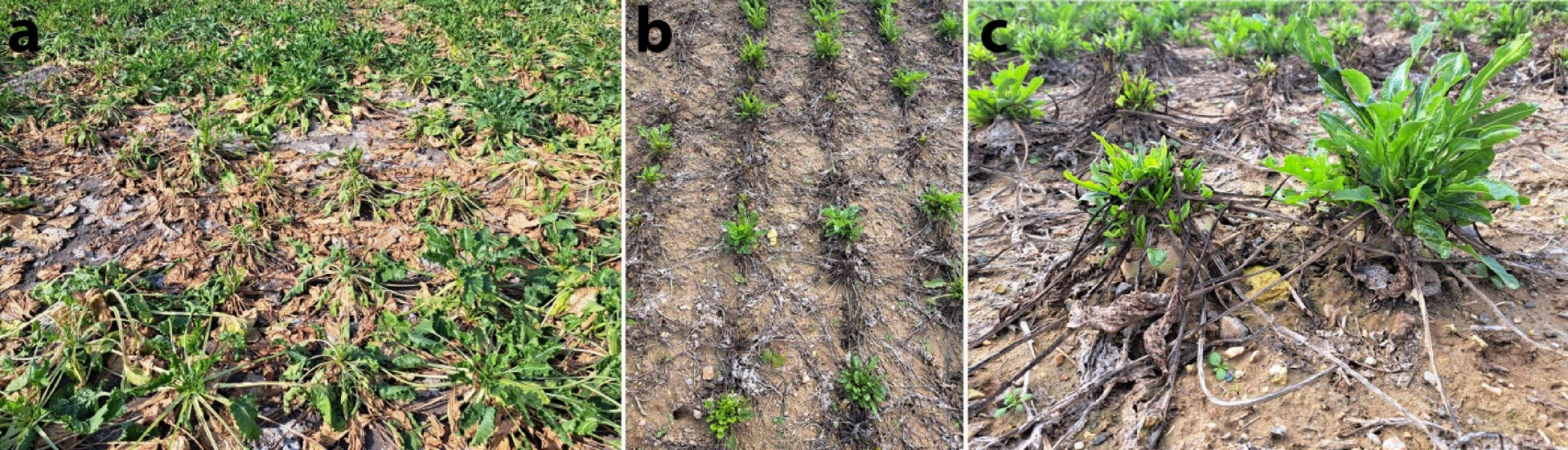
Development of symptoms in sugar beet field in Nikitsch, Austria. Plant wilting and necrosis of older leaves on September 2 (**a**); declined plants and proliferation of young leaves on October 10 (**b** and** c**).

Symptoms of SBR and/or RTD were monitored during August and September. At the end of August and in September plant wilting and necrosis of older leaves were detected in Nikitsch, but not in Rust and Katzelsdorf. The latter two locations remained without symptomatic plants, while in Nikitsch symptoms further developed during the season. These included aspecific symptoms such as leaf yellowing, necrosis, and proliferation of young leaves, whereas other symptoms were to a certain extent specifically associated either with RTD (plant decline and mild rubberiness) or SBR (asymmetry of young leaves). In October some plants declined, while proliferation of young leaves was detected on the survived ones (Fig. 4).

Ten asymptomatic sugar beet samples were randomly collected from each field in Rust and Katzelsdorf on October 4, and later evaluated for the presence of pathogens. Neither ‘*Ca.* A. phytopathogenicus’ nor ‘*Ca.* P. solani’ were detected in the tested sugar beets from Katzelsdorf. Additionally, no ‘*Ca.* A. phytopathogenicus’ was detected in sugar beets from Rust, however, ‘*Ca.* P. solani’ was found in two samples (one 16SrXII-P and one 16SrXII-A tuf-b) (Fig. 2). Fifteen sugar beet samples from Nikitsch expressing symptoms of fastidious pathogen infection were also analyzed, and ‘*Ca.* A. phytopathogenicus’ was detected in nine sugar beets, while ‘*Ca.* P. solani’ was detected in all 15 (Fig. 2). More precisely, two samples were single infected with ‘*Ca.* P. solani’ 16SrXII-P, four were mixed infected with the two phytoplasma strains, eight were mixed infected with ‘*Ca.* A. phytopathogenicus’ and ‘*Ca.* P. solani’ 16SrXII-P, while one sample contained all three pathogens.

### *Reptalus artemisiae* transmits ‘*Ca.* A. phytopathogenicus’ and ‘*Ca.* P. solani’-16SrXII-P

**Fig. 5:**
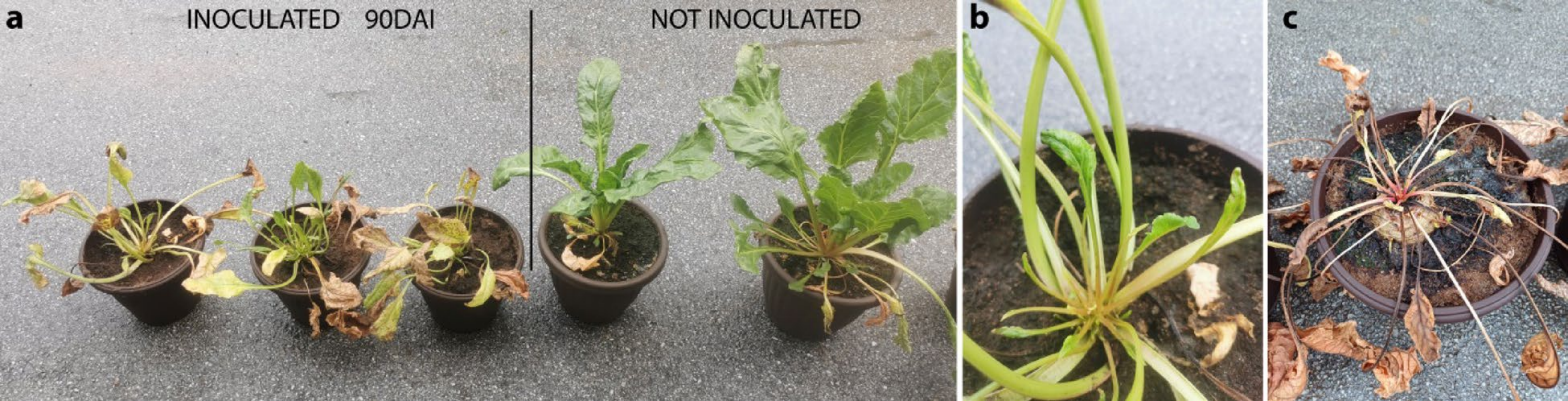
Development of symptoms in sugar beet experimentally inoculated by* R. artemisiae* from Nikitsch. Necrosis of old and proliferation of young leaves (**a**); asymmetry of young leaves (**b**) and decline of inoculated sugar beet (**c**).

Transmission trials were realized exclusively with *R. artemisiae* collected from fields in Nikitsch and Rust, as only this species was captured in sufficient numbers (Table 1). Insects used in these trials, were caught with sweeping nets on June 12 and morphologically identified, while representative number of specimens were subjected to molecular species confirmation and testing of pathogen presence (Table 1). In transmission trials 25 specimens of *R. artemisiae* were released per pot with single sugar beet plant (300 specimens in total), which was covered with a transmission cylinder to contain the insects. *R. artemisiae* specimens from each location were released into six pots with transmission cylinders (12 pots in total). After 10 days all released insects were dead. In mid-September, ~ 60 days after inoculation (60 DAI), all six experimental sugar beets exposed to *R. artemisiae* from Nikitsch developed leaf symptoms typical for both RTD and SBR - yellowing of older leaves, followed by necrosis. During the next 15 days, the older leaves declined, and proliferation of young, often asymmetric, leaves occurred (Fig. 5). Eventually all symptomatic sugar beets declined. None of the sugar beets not exposed to insects (negative control) as well as ones exposed to *R. artemisiae* collected in Rust developed any symptoms until end of the experiment and were sampled 90 DAI as asymptomatic. Molecular analyses showed that transmission trials with *R. artemisiae* collected in Nikitsch resulted in the successful transmission of both ‘*Ca*. A. phytopathogenicus’ and ‘*Ca.* P. solani’-16SrXII-P. Out of six inoculated sugar beets, four were infected with both pathogens in mixed infection, while one of the remaining two sugar beets was infected with ‘*Ca*. A. phytopathogenicus’, and the other with ‘*Ca.* P. solani’-16SrXII-P. The symptoms observed in experimentally infected sugar beets were not specific to a particular fastidious pathogen, but were instead characteristic of both, making it impossible to correlate them with a single pathogen. Insects collected in Rust did not transmit any of the tested pathogens to sugar beet.

To confirm the presence of the pathogens and test for their variability, representative strains of both pathogens were further molecularly identified and characterized through sequencing of partial 16S rRNA (Fra5/4 fragment) for ‘*Ca.* A. phytopathogenicus’ and partial *tuf* gene (fTufAY/rTufAY fragment) for ‘*Ca*. P. solani’. Partial 16S rRNA sequences of eight samples of ‘*Ca.* A. phytopathogenicus’ were obtained, with two of each detected in *R. artemisiae*, *P. leporinus* and sugar beet from fields in Nikitch, as well as two detected in sugar beets from transmission trials (one from single and one from mixed infection). Sequence analyses revealed no variability among the assessed samples and the obtained sequence was identical with the SBR-associated ‘*Ca.* A. phytopathogenicus’ type strain (AY057392) and strains detected in sugar beet in the Pannonian plain and Germany represented by HN1220/5 (MT139648).

Thirteen samples of ‘*Ca*. P. solani’ (four strains of 16SrXII-A - two from *R. artemisiae* and *P. leporinus* each, and nine of 16SrXII-P - one from sugar beet from field in Rust, and two from each of the following: *R. artemisiae*, *P. leporinus*, sugar beets from field in Nikitsch, and sugar beets inoculated in the transmission trial) were selected for sequencing of the partial *tuf* gene. Sequence analyses revealed no inter-ribosomal group variability. Notably, *tuf* sequences obtained from 16SrXII-A strains were identical to the sequence of tuf-d reference strain 429/19 (MT157234) from sugar beet in Serbia, while the sequences obtained from 16SrXII-P strains were identical to that of 16SrXII-P reference strain 916/22 (OQ717008) from sugar beet in Germany.

## Discussion

Previous studies highlighted the role of cixiid planthoppers in the transmission of ‘*Ca*. P. solani’ to sugar beet in Serbia, identifying *R. artemisiae* as the predominant vector responsible for epidemic outbreaks of RTD, with ‘*Ca*. P. solani’ prevailing over ‘*Ca*. A. phytopathogenicus’ in the Pannonian plain^6,25^. In Austria, however, ‘*Ca*. A. phytopathogenicus’ has shown greater prevalence compared to other regions of the Pannonian Plain^6^. This study sought to explore potential vector populations and their roles in transmitting ‘*Ca*. A. phytopathogenicus’ and ‘*Ca*. P. solani’, and associated diseases in Austrian sugar beet fields. However, the taxonomic status (question of the correct name) of *R. quinquecostatus* remains unresolved. Although widely used and well-defined, name *R. quinquecostatus* was rejected by Webb et al. 2013^30^ due to misinterpretation of Dufour collection specimens. Subsequently, alternative names were proposed for this taxon (*R. artemisiae* and *R. salicinus*). However, these taxa differ substantially in their host plants (wormwood and willows) and originate from regions including southern Russia (Volgograd), Crimea, and Kazakhstan and are not conspecific with the species occurring in Eastern Austria (Werner Holzinger, personal communication)^31,32^. Nevertheless, following the recent nomenclature revision^30–32^ we have adopted the name *R. artemisiae* (Becker, 1865) for the species previously referred to as *R. quinquecostatus* (Dufour) *sensu* Holzinger et al., 2003^21^ until taxonomic clarification is available.

A survey covering 33 localities across Austria’s sugar beet regions revealed striking spatial variability in cixiid abundance and disease incidence, with *R. artemisiae* dominating in most areas. In western Austria (e.g., the Linz Basin and Wachau Valley), cixiids were notably scarce. In contrast, the Vienna Basin and other parts of the Pannonian Plain exhibited higher cixiid densities and varied disease incidence, reflecting local microenvironmental influence^33^.

Temporal discrepancies between cixiid flight activity and symptom emergence complicate disease prediction and management. To address this, we conducted a detailed study at three locations with reported disease outbreaks in the previous season (2023)^6^. These locations, which included Nikitsch, Rust, and Katzelsdorf showed variable vector abundance and infection rates, which directly influenced disease occurrence. Notably, Nikitsch, with the highest cixiid abundance and its infection by the fastidious pathogens, was the only site where symptomatic sugar beet plants were observed, while no symptoms were detected at the other two locations. Such spatial variability in disease incidence is consistent with prior reports from Austria and the broader Pannonian Plain^6,33^. Molecular analyses revealed, for the first time in Austria and the Pannonian Plain, the presence of ‘*Ca*. P. solani*’* strain 16SrXII-P as dominant in Nikitsch and sporadic in Rust. The detection of this strain, previously identified as dominant on sugar beet in Germany^7,8^ underscores its epidemiological significance.

The conducted survey revealed four cixiid species as potential fastidious pathogen vectors in Austrian sugar beet fields: *H. obsoletus*, *P. leporinus*, *R. artemisiae* and *R. panzeri*. External morphological features were sufficient for reliable identification of *H. obsoletus*, *P. leporinus* and allowed identification of *R. artemisiae* only to the genus level (*Reptalus*). Specifically, *R. artemisiae* was distinguished from its congeneric species *R. panzeri* based on male genitalia, whereas females cannot be confidently distinguished morphologically. To address this limitation, a fast and reliable molecular tool has been developed and successfully applied to differentiate both male and female specimens of these two species in Italy, France, and Serbia^21,25,34–37^. Consequently, after morphological identification of all male specimens as *R. artemisiae*, we applied the previously described and validated ITS2-based molecular tool to confirm identification of 181 *Reptalus* specimens from the three locations as *R. artemisiae*. Furthermore, considering all we confirmed the identification of the cixiid species used as vector in transmission trials as *R. artemisiae* in the all 14 pathogen-carrying specimens using *COI* sequence analysis.

Transmission trials confirmed that naturally infected *R. artemisiae* populations can successfully transmit both ‘*Ca*. A. phytopathogenicus*’* and ‘*Ca*. P. solani*’* 16SrXII-P to sugar beet. While *R. artemisiae* has been established as a vector of ‘*Ca*. P. solani*’* 16SrXII-A^25,36,37^ this study is the first to demonstrate its capability to transmit ‘*Ca*. A. phytopathogenicus*’* and ‘*Ca*. P. solani*’* 16SrXII-P, as well as to induce symptoms in sugar beet typical for the diseases associated with these pathogens. Previously, it was documented that these two pathogens were transmitted only by cixiid *P. leporinus*^2,15,20^.

Despite *R. artemisiae* exhibiting lower infection rates for both ‘*Ca*. A. phytopathogenicus*’* (6.2%) and ‘*Ca*. P. solani*’* (4.7%) compared to *P. leporinus* (28% and 20%, respectively), its overwhelming abundance in Nikitsch (98.4% of the cixiid population) likely positioned it as the primary driver of pathogen epidemiology at this site. Conversely, the negligible presence of *H. obsoletus* (0.5% overall and 0.1% in Nikitsch) suggests a minimal role in disease dynamics at this location, though its involvement in other regions or seasons cannot be excluded, especially given the detection of the tuf-b strain in Rust. While *R. panzeri* appeared at six of the 33 surveyed locations in Austrian sugar beet fields, its rarity (0.3% of total cixiids) and absence from the experimental fields indicate a negligible role in transmitting ‘*Ca.* A. phytopathogenicus’ and ‘*Ca.* P. solani’ to sugar beet compared to the dominant *R. artemisiae*. The capture of *R. panzeri* in these fields is likely accidental, as a similar situation has been reported in Serbia where its presence in sugar beet fields was incidental and not linked to significant pathogen transmission, reinforcing its minor epidemiological relevance in this context^25^.

The presence of ‘*Ca*. P. solani*’* strains 16SrXII-A and -P in sugar beets in Nikitsch and also in cixiids *R. artemisiae* and *P. leporinus* suggests the same epidemiology for both ‘*Ca*. P. solani*’* strains. *R. artemisiae* has been confirmed as the main vector of ‘*Ca*. *P. solani’* 16SrXII-A, the lack of its transmission by this cixiid in the transmission trial may be due to a low infection rate of 1.6% and the relatively small number of individuals (25) used for transmission. The relatively low infection rate of *R. artemisiae*, compared to previously obtained infection rates in the Pannonian plain^25^ was revealed in this study. However, fluctuations in phytoplasma infection rates in wild populations of insect vectors, both spatially and temporally (between the seasons), are consistent with similar findings reported for *R. artemisiae* in Serbia and for *P. leporinus* in France^2,25^. Such fluctuations could be explained by interactions between the epidemiological cycle of phytoplasma and the biological cycle of insect species, as the only way insects can acquire phytoplasma is by feeding on infected reservoir plants during the larval stage.

The notable presence and higher infection rates of *P. leporinus* raise questions about its emergence or stable presence in Austrian sugar beet fields, as well as its broader epidemiological role in RTD and SBR in the Pannonian Plain. Although *P. leporinus* did not play a crucial role in epidemiology of these diseases in Austria in 2024, it may have facilitated the introduction of both ‘*Ca*. P. solani*’* 16SrXII-P and ‘*Ca*. A. phytopathogenicus*’* from Germany along the Danube Basin. This movement potentially enabled horizontal pathogen transfer to other vectors with overlapping plant hosts, such as *R. artemisiae*. Such phenomena are reminiscent of other vector-pathogen interactions, including the transfer of alder yellows/grapevine flavescence dorée phytoplasmas between *Oncopsis alni* and *Scaphoideus titanus*, or *Wolbachia* endosymbionts among phloem-feeding insects^38,39^.

The co-occurrence of *Ca. P. solani* 16SrXII-P and 16SrXII-A in sugar beet in the Pannonian Plain, coupled with their transmission by both *R. artemisiae* and *P. leporinus*, indicates that these strains are restricted neither by vector, plant host, nor geography. This adaptability complicates efforts to predict and manage disease outbreaks. Although *R. artemisiae* appears to contribute significantly to SBR epidemiology in regions lacking substantial *P. leporinus* presence in sugar beet, the impact of ‘*Ca*. A. phytopathogenicus*’* on sugar beet yield, particularly in mixed infections with ‘*Ca*. P. solani*’*, remains unclear and warrants further investigation. Results from a single growing season (2024) limit the ability to account for interannual variations in cixiid populations, pathogen prevalence, and environmental factors, necessitating multi-year studies to ensure robust conclusions. Epidemiological studies such as this are challenging, particularly when polyphagous vectors capable of transmitting multiple pathogens are involved. Vector populations must be monitored proactively and correlated with disease development, as symptomatic plants often appear after vectors have left the field. Although SBR in Austria shares similarities with the same disease in Western Europe, its transmission by *R. artemisiae*, a vector linked to RTD outbreaks in Serbia, suggests parallels in spatial and temporal disease patterns. This underscores the need for continued research into vector dynamics and pathogen transmission to inform effective management strategies.

## Methods

### Selection and monitoring of fields

The cixiid populations in 33 fields distributed across Austrian sugar beet growing areas were monitored using yellow sticky traps (23 × 36 cm; Csalomon^®^ Hungary) setup in the four corners of the fields, 2 m from the borders at a height between 120 and 140 cm. All methods applied in this study, including sampling, complied with Austrian guidelines and legislation. Access to the experimental fields was granted and permission to collect samples was obtained from AGRANA Research and Innovation Center GmbH, Tulln, Austria. Traps were maintained in sugar beet fields from mid-June until the end of August, which represents the period that covers the majority of the flight time of planthopper cixiids in central Europe^25,29,40^. Traps were replaced every 7 to 10 days, when cixiids were counted, and then removed with soft forceps from the sticky traps and subjected to morphological identification. Three fields (in the south, central and north of the sugar beet-cultivating area) were selected for pathogen assessment and transmission trials, because those fields were in the areas where both SBR and RTD had been detected previously^6^: Rust im Tullnerfeld (48^o^19’14.2’’N, 15^o^56’44.6’’E), Katzelsdorf (48^o^42’22.3’’N, 16^o^46’56.7’’E) and Nikitsch (47^o^33’05.8’’N 16^o^40’07.4’’E).

At the moment of cixiid collection for the transmission trial, 20 specimens per locality were collected using an entomological sweep net and a mouth aspirator and subjected to morphological and molecular identification, as well as assessment for the presence of ‘*Ca.* A. phytopathogenicus’ and phytoplasmas, as described later. To accurately estimate the presence of the pathogens, given the relatively low number of positive specimens, the occurrence of infield disease symptoms later in the season and successful pathogen transmission in the single plant transmission experiment, an additional 110 specimens of *R. quinquecostatus sensu* Holzinger et al.^21^ and 25 of *P. leporinus*, removed from yellow sticky traps from Nikitsch - were added to the molecular cixiid identification and pathogen assessment in insects. From Rust an additional 30 specimens of *R. artemisiae* and 10 of *P. leporinus* were removed from the yellow sticky traps and added to the molecular cixiid identification and pathogens assessment in insects. At the end of the season, from October 10 to October 12, 10 asymptomatic sugar beet samples were randomly collected from Rust and Katzelsdorf each, while 15 sugar beets with typical symptoms of fastidious pathogen infection were collected from Nikitsch for pathogens assessment.

### Nucleic acid extraction of plants and insects

Total nucleic acids extraction from plants and individual insects was done following the CTAB protocols^20,41^. The isolated nucleic acids were resuspended in TE buffer and stored at -20 °C until further analysis.

### Insect identification

A stereo microscope was used to initially identify all collected cixiids as *P. leporinus*, *H. obsoletus* or, in the case of *Reptalus* sp., at the genus level according to external morphological features (by the size of the body, five distinct longitudinal carinae on the mesonotum and two transverse keels on the head). Male specimens were determined as *R. artemisiae* according to male genitalia i.e. morphology of the anal tube and presence of a left orientated process (Supplementary Fig. S1, S2)^21^. Although this species has been reported under the long-time used and well defined name ‘*R. quinquecostatus’* as vector of phytoplasmas on sugar beet, as well as regarding other plant diseases in various publications^25,32,36^ the question of the correct name of the *Reptalus* species of our study is still unresolved. Following recent nomenclatural revisions^30–32^ the use of the name ‘*R. quinquecostatus’* is now considered incorrect. Therefore, until taxonomic clarification is achieved, we refer to this species as *R. artemisiae*, previously reported as *R. quinquecostatus sensu* Holzinger et al. 2003^21^. Since the female specimens of *Reptalus* spp. could not be reliably distinguished morphologically, for identification at the species level, *Reptalus* sp. specimens from the three fields selected for pathogen assessment and transmission trials were subjected to molecular identification based on differences in the length of internal transcribed spacer 2 (ITS2) amplicons^34^. Primer pair ITS2fw/ITS2rv^42^ was used for amplification of ITS2 of the ribosomal DNA in a 25-µL final reaction volume containing 0.5 µl of template DNA (isolated as described above), 1 x PCR Master Mix (Thermo Scientific, Vilnius, Lithuania), and 0.4 µM of each primer under previously described thermal conditions^34^. Six microliters of PCR products were separated in a 1% agarose gel, stained with ethidium bromide and visualized with a UV transilluminator. To confirm identification of insect species, insects carrying pathogens were subjected to amplification of partial Cytochrome c Oxidase subunit 1 (*COI*) gene using C1J-2195/TL2-N-3014 (~ 800 bp) and Cl-J-1751/Calvin (~ 1 Kbp) primer pairs^43–45^ and sequence analyses. In total, all 14 *R. artemisiae* specimens carrying any pathogen (‘*Ca.* A. phytopathogenicus’, ‘*Ca*. P. solani*’* strains 16SrXII-A and -P), and four *P. leporinus* specimens, with two carrying each phytoplasma strain in single infection and two in mixed with ‘*Ca.* A. phytopathogenicus’, were subjected to *COI* sequencing. The reaction mix and visualization were as described for ITS2 amplification, while thermal conditions were as described previously^43,45^. All PCR products were sequenced in both directions by a commercial service (Macrogen Inc., Seoul, Korea), using the primers previously applied in their amplification. The obtained *COI* sequences were assembled with Pregap4 from the Staden Package^46^ manually inspected and aligned with publicly available cixiids *COI* sequences using ClustalX, under MEGA version X^47,48^ for phylogenetic analyses. The obtained partial *COI* gene sequences of representative genotypes of *R. artemisiae* and *P. leporinus* were deposited in the NCBI GenBank under acc. numbers provided in Fig. 3. Evolutionary history based on the *COI* sequences obtained in this study and 15 publicly available cixiid species was inferred using the maximum-likelihood (ML) method (MEGA X) and best-fit substitution model. Initial trees for the heuristic search were obtained automatically by applying the Neighbor-Join and BioNJ algorithms. The closest species *Catonia carolina* (Achilidae) was used as an outgroup taxon to root the trees. To estimate the statistical significance of the inferred clades, 1,000 bootstraps were performed.

### Phytoplasma assessment in plants and insects

The presence of phytoplasmas was assessed in all samples by nested PCR system targeting the 16S rDNA of phytoplasmas. Direct PCR assays with P1/P7 primer pair^49,50^ were followed by nested PCR with R16F2n/R2 under previously described conditions^51^. Each 25 µL PCR mix contained 20 ng of template DNA, 1× PCR Master Mix and 0.4 µM of each primer. *‘Ca*. P. solani’ strain 284/09 ^52^ was used as a positive control, whereas reaction mixes lacking template DNA were employed as negative controls. For the nested PCR, 0.5 µL of direct PCR amplicon was used as a template. Six microliters of PCR products were separated and visualized as described above.

For phytoplasma identification and characterization, phytoplasma-positive samples were subjected to *tuf* gene analyses. Direct PCR assays with Tuf1-f1/Tuf1-r1 were followed by nested PCR with fTufAY/rTufAY under previously described conditions^53–55^. Each 25 µL PCR mix, as well as positive and negative controls, were prepared as described above. For the nested PCR, 0.5 µL of direct PCR amplicon was used as a template. PCR products were separated and visualized as described above. For tuf-type differentiation, all samples were subjected to RFLP analyses of the *tuf* gene as described previously^1^. Products of the RFLP reaction were separated in an 8% polyacrylamide gel, stained and visualized as described above. Thirteen phytoplasma-positive samples were selected and subjected to tuf sequencing in both directions with primers applied for amplification (fTufAY/rTufAY) - four *‘Ca*. P. solani’ 16SrXII-A (two from *R. artemisiae* and *P. leporinus* each) and nine *‘Ca*. P. solani’ 16SrXII-P (one from sugar beet from field in Rust, and two from each of the following: *R. artemisiae*, *P. leporinus*, sugar beets from field in Nikitsch, and sugar beets inoculated in the transmission trial). The obtained sequences of *‘Ca*. P. solani’ partial *tuf* gene were assembled using Pregap4 from the Staden Package^46^ manually inspected and aligned with publicly available sequences using ClustalX, under MEGA version X^47,48^.

### Proteobacterium assessment in plants and insects

The presence of *‘Ca*. A. phytopathogenicus’ in insect samples was assessed using the Fra5/4 primer pair, which is specific to the 16S rRNA gene of ‘*Ca*. A. phytopathogenicus’. The reaction mix and conditions were applied as previously described^56,57^.

The presence of *‘Ca*. A. phytopathogenicus’ in plant samples was initially assessed using the TaqMan real-time PCR (qPCR) protocol targeting the *hsp20* gene (small heat shock protein) with previously described cycling parameters and reaction mix^58^. Each reaction included a DNA-free blank assay, a negative control corresponding to an asymptomatic sugar beet, and a positive control of ‘*Ca*. A. phytopathogenicus’, strain HN1220/5 ^33^. All qPCRs were performed in the Magnetic Induction Cycler, Mic (Bio Molecular Systems, Upper Coomera, Australia). Data evaluation for all qPCR assays was performed using micPCR© software Version 2.6.4 (Bio Molecular Systems, Upper Coomera, Australia). Samples were considered positive if they produced an amplification curve with exponential growth with a C_q_ value of < 35. For confirmation and identification, all positive samples were subjected to the Fra5/4 PCR reaction.

Eight Fra5/4 PCR positive samples were selected (two from each of the following: *R. artemisiae*, *P. leporinus*, sugar beets from field in Nikitsch, and sugar beets inoculated in the transmission trial) and subjected to sequencing in both directions with primers applied for amplification. The obtained sequences of *‘Ca*. A. phytopathogenicus’ partial 16S rRNA gene were assembled, manually inspected and aligned with publicly available sequences as described above.

### Transmission trials

Naturally infected populations of cixiid vector *R. artemisiae* from Rust and Nikitsch were used in transmission trials performed on individual sugar beet seedlings, as this species was the only cixiid present at these two locations on July 12, at the time of sweep netting. Two cixiid species (*R. artemisiae* and *P. leporinus*) were present in small numbers at the third location, Katzelsdorf, but were not used in the trial, as few were caught with the sweep net. Identification of cixiids enrolled in the transmission trial as *R. artemisiae* and fastidious pathogen presence was verified on representative number of individuals via morphological and molecular methods. Plants for the transmission trial were seeded, one per pot in pathogen-free soil and maintained in an insect-proof environment in a climate chamber at 25 ± 2 °C (16/8 h light/dark period). A transparent plastic cylinder (25 cm height and 9 cm diameter, perfectly matching each pot) covered with a mesh for ventilation was placed on each pot and fixed using crepe tape. Planthoppers were collected and 25 were transferred to each transmission cylinder. Six sugar beets were used to test pathogen transmission via planthoppers from each of the two locations. The planting pots with the cylinders were placed in a climate chamber with controlled conditions. Five sugar beets not exposed to insects were included in the experiment as negative controls. All plants were monitored weekly for symptom development and were sampled 90 DAI. The insects were left on the plants until they died. When all insects in a pot were dead 10 days after release, the cylinders were removed, and all plants were placed in a climate chamber and monitored for symptoms. After a month, the sugar beet plants were transplanted into bigger containers.

## Electronic supplementary material

Below is the link to the electronic supplementary material.

Supplementary Material 1

## Data Availability

All data generated or analyzed during this study are included in this published article (and its Supplementary Information files). The sequence data have been deposited in NCBI GenBank (www.ncbi.nlm.nih.gov/genbank) with the accession codes used in Fig. 3.
