# Supplementary material for: Dominant cixiid vector and transmission of ‘Candidatus Arsenophonus phytopathogenicus’ and ‘Candidatus Phytoplasma solani’-related strain 16SrXII-P in sugar beet in Austria
Source: Sci Rep. 2025 Jul 2;15:22526. doi: 10.1038/s41598-025-07035-0 (PMC12218963; doi:10.1038/s41598-025-07035-0)

**Transmission of ‘*Ca.* Arsenophonus phytopathogenicus’ and ‘*Ca.* Phytoplasma solani’-Related Strain 16SrXII-P by *Reptalus quinquecostatus* to Sugar Beet**

**Christoph Kreitzer, Jelena Stepanović, Nikola Stanojević, Anna Rohringer, Seiter Marion, Emil Rekanović, and Bojan Duduk**

Supplementary Table S1. List of the 33 locations in Austria surveyed in the study and cixiid species caught on yellow sticky traps. Localities of three experimental fields selected for a comprehensive cixiid survey, testing of pathogen presence in insect, and transmission trials are highlighted in bold. *Yellow sticky traps and location where *R. panzeri* is caught are marked with asterisk in “Total cixiid” column

| **Location** | **GPS** | **Date of trap collection** | **Number of specimens** | | | |
| --- | --- | --- | --- | --- | --- | --- |
|  |  |  | ***Reptalus***  ***artemisiae*** | ***Pentastiridius***  ***leporinus*** | ***Hyalesthes***  ***obsoletus*** | **Total cixiid*** |
| Altlichtenwarth | 48.6567840665241  16.8283960623167 | 2024-06-03 | 0 | 1 | 0 | 1 |
|  |  | 2024-06-10 | 2 | 0 | 2 | 4 |
|  |  | 2024-06-17 | 10 | 0 | 0 | 10 |
|  |  | 2024-06-24 | 2 | 0 | 0 | 2 |
|  |  | 2024-07-01 | 5 | 0 | 0 | 5 |
|  |  | 2024-07-08 | 6 | 0 | 0 | 6 |
|  |  | 2024-07-15 | 8 | 0 | 0 | 8 |
| Deutschkreutz | 47.6021965006971  16.6406067353777 | 2024-06-10 | 0 | 0 | 0 | 0 |
|  |  | 2024-06-17 | 0 | 0 | 1 | 1 |
|  |  | 2024-06-24 | 3 | 0 | 1 | 4 |
|  |  | 2024-07-08 | 12 | 1 | 0 | 13 |
|  |  | 2024-07-15 | 8 | 0 | 0 | 8 |
|  |  | 2024-07-22 | 2 | 0 | 0 | 2 |
|  |  | 2024-08-05 | 0 | 0 | 0 | 0 |
| Drösing | 48.5550753034933  16.9179249137404 | 2024-06-10 | 1 | 5 | 0 | 6 |
|  |  | 2024-06-17 | 3 | 2 | 0 | 5 |
|  |  | 2024-06-24 | 1 | 1 | 0 | 2 |
|  |  | 2024-07-08 | 11 | 0 | 0 | 12* |
|  |  | 2024-07-15 | 20 | 0 | 0 | 21* |
|  |  | 2024-07-29 | 14 | 2 | 0 | 17* |
|  |  | 2024-08-05 | 17 | 0 | 0 | 17 |
| Eisenstadt | 47.821065310419  16.4776838211537 | 2024-06-03 | 0 | 0 | 0 | 0 |
|  |  | 2024-06-10 | 1 | 0 | 0 | 1 |
|  |  | 2024-06-17 | 1 | 0 | 0 | 1 |
|  |  | 2024-06-24 | 1 | 0 | 0 | 1 |
|  |  | 2024-07-08 | 8 | 0 | 1 | 9 |
|  |  | 2024-07-15 | 4 | 0 | 0 | 4 |
|  |  | 2024-07-22 | 1 | 0 | 0 | 1 |
| Götzendorf | 48.0190224180906  16.5440356110918 | 2024-06-17 | 0 | 0 | 0 | 0 |
|  |  | 2024-06-17 | 3 | 0 | 2 | 5 |
|  |  | 2024-06-24 | 8 | 5 | 0 | 13 |
|  |  | 2024-07-08 | 7 | 1 | 0 | 9* |
|  |  | 2024-07-15 | 12 | 0 | 0 | 12 |
|  |  | 2024-07-22 | 7 | 0 | 0 | 8* |
|  |  | 2024-08-05 | 5 | 0 | 0 | 5 |
| Grafensulz | 48.556762459666  16.4304557016058 | 2024-06-17 | 26 | 19 | 0 | 45 |
|  |  | 2024-06-24 | 51 | 10 | 1 | 62 |
|  |  | 2024-07-01 | 149 | 38 | 0 | 187 |
|  |  | 2024-07-08 | 221 | 6 | 2 | 229 |
|  |  | 2024-07-15 | 114 | 0 | 1 | 115 |
|  |  | 2024-07-29 | 41 | 1 | 0 | 42 |
|  |  | 2024-08-05 | 35 | 0 | 0 | 35 |
| Großhöflein/  Eisenstadt | 47.8288781825259  16.508830896187 | 2024-06-03 | 0 | 0 | 0 | 0 |
|  |  | 2024-06-10 | 0 | 0 | 0 | 0 |
|  |  | 2024-06-17 | 5 | 0 | 0 | 5 |
|  |  | 2024-06-24 | 4 | 0 | 2 | 6 |
|  |  | 2024-07-08 | 6 | 0 | 0 | 6 |
|  |  | 2024-07-15 | 9 | 1 | 1 | 11 |
|  |  | 2024-07-22 | 9 | 0 | 0 | 9 |
| Großkrut | 48.647013122087  16.726939172425 | 2024-06-03 | 0 | 0 | 0 | 0 |
|  |  | 2024-06-10 | 3 | 2 | 0 | 5 |
|  |  | 2024-06-17 | 11 | 1 | 1 | 13 |
|  |  | 2024-06-24 | 6 | 1 | 0 | 7 |
|  |  | 2024-07-01 | 22 | 1 | 0 | 23 |
|  |  | 2024-07-08 | 30 | 0 | 1 | 31 |
|  |  | 2024-07-15 | 4 | 0 | 0 | 4 |
| Guntersdorf | 48.6474355354443  16.0328038522914 | 2024-06-17 | 1 | 0 | 0 | 1 |
|  |  | 2024-06-24 | 1 | 2 | 0 | 3 |
|  |  | 2024-07-01 | 1 | 4 | 0 | 5 |
|  |  | 2024-07-08 | 1 | 4 | 0 | 5 |
|  |  | 2024-07-15 | 1 | 0 | 0 | 1 |
|  |  | 2024-07-22 | 0 | 1 | 0 | 1 |
|  |  | 2024-08-05 | 1 | 0 | 0 | 1 |
| Hirm | 47.7882737695622  16.4439007929122 | 2024-06-03 | 0 | 2 | 0 | 2 |
|  |  | 2024-06-10 | 0 | 23 | 1 | 24 |
|  |  | 2024-06-17 | 0 | 16 | 6 | 22 |
|  |  | 2024-06-24 | 2 | 17 | 0 | 19 |
|  |  | 2024-07-08 | 6 | 21 | 4 | 31 |
|  |  | 2024-07-15 | 0 | 7 | 0 | 7 |
|  |  | 2024-07-22 | 0 | 0 | 0 | 0 |
| Höflein | 48.0785353060513  16.8002495608479 | 2024-06-24 | 1 | 0 | 0 | 1 |
|  |  | 2024-07-01 | 2 | 0 | 0 | 2 |
|  |  | 2024-07-08 | 0 | 0 | 0 | 0 |
|  |  | 2024-07-15 | 1 | 0 | 0 | 1 |
|  |  | 2024-07-22 | 0 | 0 | 0 | 0 |
|  |  | 2024-07-29 | 1 | 0 | 0 | 1 |
|  |  | 2024-06-17 | 0 | 0 | 0 | 0 |
| Illmitz | 47.8221693694941  16.8443024458222 | 2024-06-03 | 0 | 0 | 0 | 0 |
|  |  | 2024-06-24 | 0 | 0 | 0 | 0 |
|  |  | 2024-06-17 | 1 | 0 | 0 | 1 |
|  |  | 2024-07-08 | 1 | 0 | 0 | 1 |
|  |  | 2024-07-15 | 0 | 0 | 0 | 0 |
|  |  | 2024-07-22 | 0 | 0 | 0 | 0 |
|  |  | 2024-06-10 | 0 | 0 | 0 | 0 |
| **Katzelsdorf** | 48.7069934818149  16.7827206481296 | 2024-06-03 | 0 | 0 | 0 | 0 |
|  |  | 2024-06-10 | 0 | 2 | 0 | 2 |
|  |  | 2024-06-17 | 1 | 0 | 0 | 1 |
|  |  | 2024-06-24 | 1 | 24 | 2 | 27 |
|  |  | 2024-07-01 | 2 | 4 | 0 | 6 |
|  |  | 2024-07-08 | 4 | 0 | 0 | 4 |
|  |  | 2024-07-15 | 2 | 1 | 1 | 4 |
| Katzelsdorf2 | 48.6814932142889  16.7806340959708 | 2024-06-24 | 3 | 1 | 0 | 4 |
|  |  | 2024-07-01 | 4 | 1 | 0 | 5 |
|  |  | 2024-07-08 | 5 | 0 | 1 | 6 |
|  |  | 2024-06-17 | 0 | 2 | 0 | 2 |
|  |  | 2024-07-15 | 4 | 0 | 0 | 4 |
|  |  | 2024-07-29 | 0 | 0 | 0 | 0 |
|  |  | 2024-08-05 | 0 | 0 | 0 | 0 |
| Kirchberg-Thening | 48.2503372644933  14.1612312298713 | 2024-06-24 | 0 | 2 | 2 | 4 |
|  |  | 2024-07-08 | 0 | 2 | 0 | 2 |
|  |  | 2024-08-05 | 1 | 0 | 0 | 1 |
|  |  | 2024-07-15 | 0 | 0 | 0 | 0 |
| Kopfstetten/  Eckartsau | 48.1503551685876  16.8082486985322 | 2024-06-17 | 0 | 0 | 1 | 1 |
|  |  | 2024-06-24 | 7 | 2 | 3 | 12 |
|  |  | 2024-07-01 | 4 | 0 | 0 | 4 |
|  |  | 2024-07-08 | 2 | 2 | 0 | 4 |
|  |  | 2024-07-15 | 1 | 0 | 0 | 1 |
|  |  | 2024-07-22 | 6 | 0 | 0 | 6 |
|  |  | 2024-08-05 | 0 | 0 | 0 | 0 |
| Mönchhof | 47.9164765059081  16.9691459735854 | 2024-06-03 | 1 | 1 | 1 | 3 |
|  |  | 2024-06-24 | 13 | 1 | 6 | 20 |
|  |  | 2024-06-17 | 1 | 1 | 2 | 4 |
|  |  | 2024-07-08 | 25 | 9 | 0 | 34 |
|  |  | 2024-06-10 | 4 | 1 | 2 | 7 |
|  |  | 2024-07-15 | 4 | 0 | 0 | 4 |
|  |  | 2024-07-22 | 14 | 1 | 1 | 16 |
| Moosham | 48.2599149358316  13.2590746065137 | 2024-06-24 | 0 | 1 | 0 | 1 |
|  |  | 2024-07-08 | 0 | 0 | 0 | 0 |
|  |  | 2024-08-05 | 0 | 0 | 0 | 0 |
|  |  | 2024-08-26 | 0 | 0 | 0 | 0 |
|  |  | 2024-07-15 | 0 | 0 | 0 | 0 |
| **Nikitsch** | 47.55190395379  16.6694006592848 | 2024-06-10 | 146 | 0 | 1 | 147 |
|  |  | 2024-06-17 | 332 | 2 | 0 | 334 |
|  |  | 2024-06-24 | 336 | 3 | 0 | 339 |
|  |  | 2024-07-08 | 410 | 15 | 0 | 425 |
|  |  | 2024-07-15 | 378 | 2 | 0 | 380 |
|  |  | 2024-07-22 | 129 | 1 | 0 | 130 |
| Obersiebenbrunn | 48.2701746296446  16.6795202222149 | 2024-06-17 | 0 | 1 | 0 | 1 |
|  |  | 2024-06-24 | 2 | 9 | 0 | 11 |
|  |  | 2024-07-01 | 0 | 0 | 1 | 2* |
|  |  | 2024-07-08 | 0 | 1 | 1 | 2 |
|  |  | 2024-07-15 | 0 | 0 | 0 | 0 |
|  |  | 2024-07-22 | 0 | 0 | 0 | 0 |
|  |  | 2024-08-05 | 0 | 0 | 0 | 0 |
| Ottenthal | 48.7650475988947  16.5932678228664 | 2024-06-24 | 1 | 0 | 1 | 3* |
|  |  | 2024-06-17 | 1 | 1 | 1 | 3 |
|  |  | 2024-07-01 | 28 | 12 | 1 | 42* |
|  |  | 2024-07-08 | 18 | 0 | 0 | 19* |
|  |  | 2024-07-15 | 36 | 1 | 0 | 38* |
|  |  | 2024-07-29 | 6 | 0 | 0 | 6 |
|  |  | 2024-08-05 | 3 | 0 | 0 | 3 |
| Pamhagen | 47.6954396672039  16.9436689466462 | 2024-06-03 | 5 | 0 | 0 | 5 |
|  |  | 2024-06-24 | 46 | 1 | 0 | 47 |
|  |  | 2024-06-17 | 42 | 1 | 2 | 45 |
|  |  | 2024-07-08 | 35 | 0 | 0 | 35 |
|  |  | 2024-07-15 | 10 | 0 | 0 | 10 |
|  |  | 2024-07-22 | 3 | 0 | 0 | 3 |
|  |  | 2024-06-10 | 16 | 1 | 0 | 17 |
| Patzmannsdorf | 48.6390203738476  16.2675416265779 | 2024-06-17 | 3 | 2 | 0 | 5 |
|  |  | 2024-06-24 | 8 | 7 | 0 | 15 |
|  |  | 2024-07-01 | 23 | 11 | 0 | 34 |
|  |  | 2024-07-08 | 15 | 9 | 0 | 24 |
|  |  | 2024-07-15 | 21 | 0 | 0 | 21 |
|  |  | 2024-07-22 | 3 | 1 | 0 | 4 |
|  |  | 2024-08-05 | 1 | 0 | 0 | 1 |
| Podersdorf/  Frauenkirchen | 47.848779734453  16.8830406525035 | 2024-06-03 | 0 | 0 | 0 | 0 |
|  |  | 2024-06-24 | 2 | 0 | 0 | 2 |
|  |  | 2024-06-17 | 2 | 0 | 0 | 2 |
|  |  | 2024-06-10 | 1 | 0 | 0 | 1 |
|  |  | 2024-07-08 | 9 | 0 | 0 | 9 |
|  |  | 2024-07-15 | 3 | 0 | 0 | 3 |
|  |  | 2024-07-22 | 2 | 0 | 0 | 2 |
| Ringendorf | 48.4970814649428  16.1996145732782 | 2024-06-17 | 1 | 0 | 0 | 2* |
|  |  | 2024-06-24 | 0 | 6 | 0 | 7* |
|  |  | 2024-07-01 | 0 | 0 | 0 | 0 |
|  |  | 2024-07-08 | 6 | 0 | 0 | 6 |
|  |  | 2024-07-15 | 0 | 0 | 0 | 0 |
|  |  | 2024-07-22 | 0 | 0 | 0 | 0 |
|  |  | 2024-08-05 | 0 | 0 | 0 | 0 |
| **Rust** | 48.3205569011673  15.9454189139465 | 2024-06-17 | 57 | 7 | 2 | 66 |
|  |  | 2024-06-24 | 104 | 3 | 0 | 107 |
|  |  | 2024-07-01 | 150 | 8 | 1 | 159 |
|  |  | 2024-07-08 | 91 | 7 | 1 | 99 |
|  |  | 2024-07-15 | 41 | 1 | 0 | 42 |
|  |  | 2024-07-22 | 54 | 0 | 0 | 54 |
|  |  | 2024-08-05 | 12 | 0 | 0 | 12 |
| Sankt Peter am Hart | 48.242239654842  13.082326472385 | 2024-08-05 | 0 | 0 | 0 | 0 |
|  |  | 2024-07-22 | 0 | 0 | 0 | 0 |
|  |  | 2024-07-01 | 0 | 1 | 0 | 1 |
| Schranawand | 47.9763119528702  16.4590424577991 | 2024-06-17 | 0 | 0 | 0 | 0 |
|  |  | 2024-07-01 | 5 | 1 | 1 | 7 |
|  |  | 2024-07-08 | 9 | 0 | 0 | 9 |
|  |  | 2024-07-15 | 6 | 0 | 0 | 6 |
|  |  | 2024-07-22 | 10 | 0 | 1 | 11 |
|  |  | 2024-08-05 | 3 | 0 | 0 | 3 |
|  |  | 2024-06-17 | 1 | 0 | 0 | 1 |
| Siegendorf | 47.7772830648685  16.5137098827249 | 2024-06-03 | 0 | 0 | 0 | 0 |
|  |  | 2024-06-17 | 7 | 0 | 1 | 8 |
|  |  | 2024-06-24 | 21 | 0 | 2 | 23 |
|  |  | 2024-07-08 | 61 | 1 | 0 | 62 |
|  |  | 2024-07-15 | 104 | 0 | 0 | 104 |
|  |  | 2024-07-22 | 41 | 0 | 0 | 41 |
|  |  | 2024-06-10 | 1 | 0 | 0 | 1 |
| Tadten | 47.7585601613375  17.0113755458457 | 2024-06-03 | 0 | 0 | 5 | 5 |
|  |  | 2024-06-24 | 6 | 0 | 0 | 6 |
|  |  | 2024-06-17 | 10 | 2 | 0 | 12 |
|  |  | 2024-07-08 | 12 | 0 | 0 | 12 |
|  |  | 2024-07-15 | 4 | 0 | 0 | 4 |
|  |  | 2024-07-22 | 2 | 0 | 0 | 2 |
|  |  | 2024-06-10 | 5 | 3 | 0 | 8 |
| Velm-Götzendorf | 48.4880672723683  16.7809152270905 | 2024-06-24 | 0 | 0 | 0 | 1* |
|  |  | 2024-06-17 | 0 | 0 | 0 | 0 |
|  |  | 2024-07-08 | 9 | 0 | 0 | 9 |
|  |  | 2024-07-15 | 2 | 0 | 0 | 2 |
|  |  | 2024-07-29 | 0 | 0 | 0 | 0 |
|  |  | 2024-08-05 | 1 | 0 | 0 | 1 |
| Wildendürnbach | 48.7620703577523  16.4675585286649 | 2024-06-24 | 7 | 2 | 0 | 9 |
|  |  | 2024-07-01 | 15 | 2 | 0 | 17 |
|  |  | 2024-07-08 | 9 | 0 | 0 | 9 |
|  |  | 2024-07-15 | 2 | 0 | 0 | 2 |
|  |  | 2024-07-22 | 7 | 0 | 0 | 7 |
|  |  | 2024-08-05 | 0 | 4 | 0 | 4 |
|  |  | 2024-06-17 | 3 | 1 | 0 | 4 |
| Wulzeshofen | 48.7021590289509  16.3002637209132 | 2024-06-24 | 4 | 2 | 0 | 6 |
|  |  | 2024-07-01 | 11 | 6 | 0 | 17 |
|  |  | 2024-07-08 | 6 | 0 | 0 | 6 |
|  |  | 2024-07-15 | 9 | 0 | 0 | 9 |
|  |  | 2024-07-22 | 6 | 0 | 0 | 6 |
|  |  | 2024-08-05 | 2 | 2 | 0 | 4 |
|  |  | 2024-06-17 | 0 | 10 | 0 | 10 |
|  |  | **Total** | **4052** | **403** | **71** | **4539** |

Supplementary Figure S1. *Reptalus artemisiae* (referred before as *Reptalus quinquecostatus* *sensu* Holzinger et al. 2003) on sugar beet leaf in Nikitsch, Austria


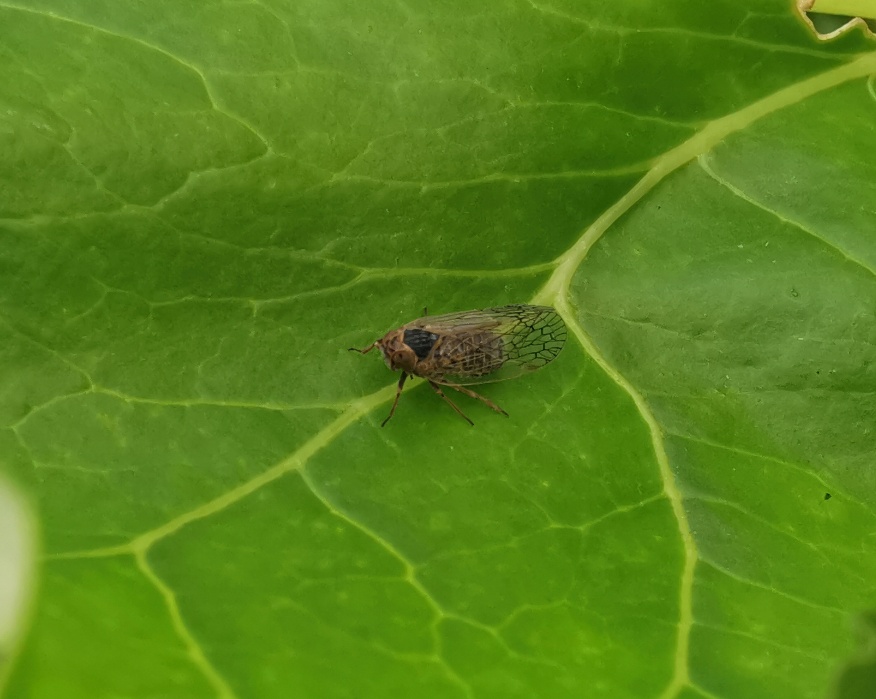


Supplementary Figure S2. Anal tubes of *R. artemisiae* (referred before as *Reptalus quinquecostatus sensu* Holzinger et al. 2003) (a, b, c) of specimens from population used in transmission trials with visible left orientated process pointed by arrows and anal tube of *R. panzeri* (d) without process.


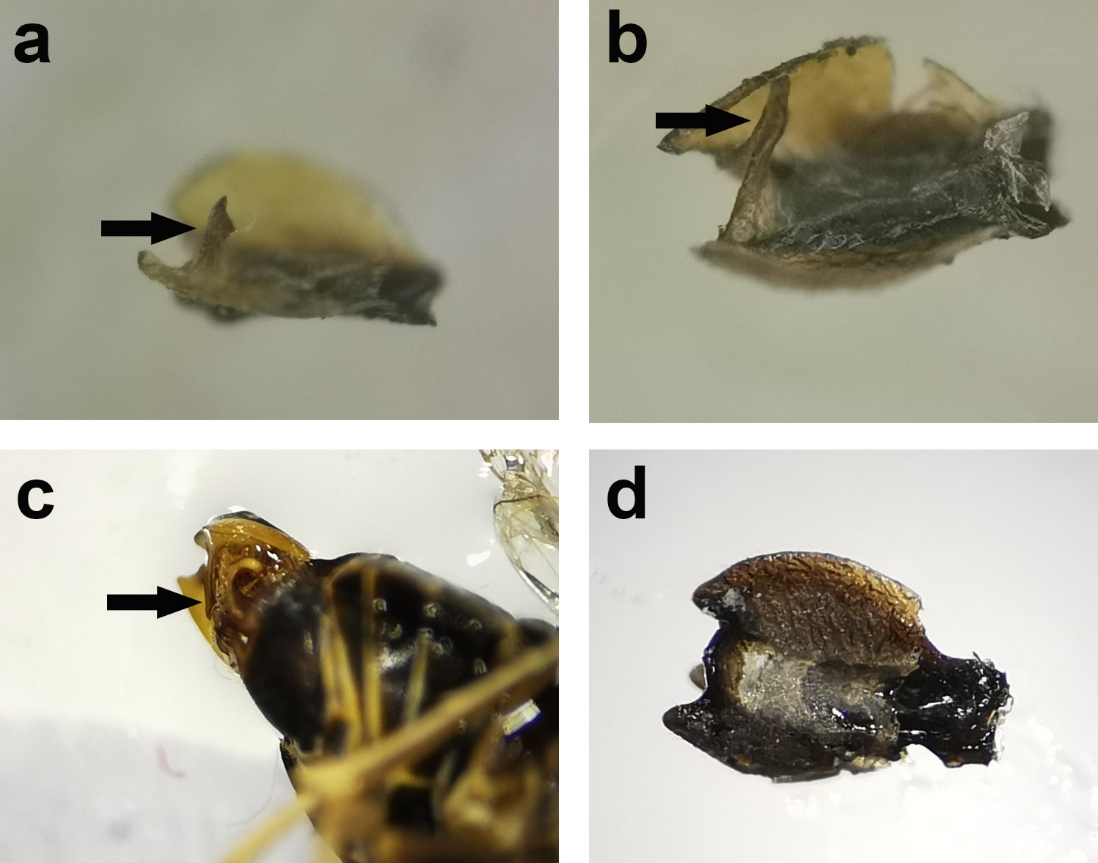

Supplement: Supplementary file 1 — Supplementary Material 1 [file 41598_2025_7035_MOESM1_ESM.docx]
